# Spike Timing Rigidity Is Maintained in Bursting Neurons under Pentobarbital-Induced Anesthetic Conditions

**DOI:** 10.3389/fncir.2016.00086

**Published:** 2016-11-14

**Authors:** Risako Kato, Masanori Yamanaka, Eiko Yokota, Noriaki Koshikawa, Masayuki Kobayashi

**Affiliations:** ^1^Department of Pharmacology, School of Dentistry, Nihon UniversityChiyoda, Japan; ^2^Division of Oral and Craniomaxillofacial Research, Dental Research Center, School of Dentistry, Nihon UniversityChiyoda, Japan; ^3^Department of Physics, College of Science and Technology, Nihon UniversityChiyoda, Japan; ^4^Department of Anesthesiology, School of Dentistry, Nihon UniversityChiyoda, Japan; ^5^Molecular Dynamics Imaging Unit, RIKEN Center for Life Science TechnologiesKobe, Japan

**Keywords:** insular cortex, interspike interval, unfolding transformation, GABA_A_ receptor, barbiturate

## Abstract

Pentobarbital potentiates γ-aminobutyric acid (GABA)-mediated inhibitory synaptic transmission by prolonging the open time of GABA_A_ receptors. However, it is unknown how pentobarbital regulates cortical neuronal activities via local circuits *in vivo*. To examine this question, we performed extracellular unit recording in rat insular cortex under awake and anesthetic conditions. Not a few studies apply time-rescaling theorem to detect the features of repetitive spike firing. Similar to these methods, we define an average spike interval locally in time using random matrix theory (RMT), which enables us to compare different activity states on a universal scale. Neurons with high spontaneous firing frequency (>5 Hz) and bursting were classified as HFB neurons (*n* = 10), and those with low spontaneous firing frequency (<10 Hz) and without bursting were classified as non-HFB neurons (*n* = 48). Pentobarbital injection (30 mg/kg) reduced firing frequency in all HFB neurons and in 78% of non-HFB neurons. RMT analysis demonstrated that pentobarbital increased in the number of neurons with repulsion in both HFB and non-HFB neurons, suggesting that there is a correlation between spikes within a short interspike interval (ISI). Under awake conditions, in 50% of HFB and 40% of non-HFB neurons, the decay phase of normalized histograms of spontaneous firing were fitted to an exponential function, which indicated that the first spike had no correlation with subsequent spikes. In contrast, under pentobarbital-induced anesthesia conditions, the number of non-HFB neurons that were fitted to an exponential function increased to 80%, but almost no change in HFB neurons was observed. These results suggest that under both awake and pentobarbital-induced anesthetized conditions, spike firing in HFB neurons is more robustly regulated by preceding spikes than by non-HFB neurons, which may reflect the GABA_A_ receptor-mediated regulation of cortical activities. Whole-cell patch-clamp recording in the IC slice preparation was performed to compare the regularity of spike timing between pyramidal and fast-spiking (FS) neurons, which presumably correspond to non-HFB and HFB neurons, respectively. Repetitive spike firing of FS neurons exhibited a lower variance of ISI than pyramidal neurons both in control and under application of pentobarbital, supporting the above hypothesis.

## Introduction

The cerebral cortex has been a principal target in cognitive neuroscience because it processes higher brain functions such as cognition, voluntary movement, prediction, and learning and memory. General anesthetics modulate the neural activities of glutamatergic excitatory and GABAergic inhibitory neurons (Patel and Chapin, 1990) and the synaptic activities in the cerebral cortex (el-Beheiry and Puil, 1989; Wakita et al., 2013). As a result, the excitatory and inhibitory balance is changed, and the brain state transitions from conscious to unconscious (Vizuete et al., 2012; Taub et al., 2013).

Pentobarbital, a short-acting barbiturate, potentiates GABAergic inhibition by prolonging the duration of GABA_A_ receptors’ opening (Thompson et al., 1996); as a result, it reduces the excitatory outputs from cortical local circuits. Moreover, GABAergic interneurons project to neighboring interneurons and/or themselves (Bacci et al., 2003; Koyanagi et al., 2010). Therefore, the pentobarbital-induced potentiation of GABAergic currents may induce disinhibition, facilitating cortical outputs. To test these contradictory hypotheses, it is critical to understand the pentobarbital-induced modulation of excitatory and inhibitory neurons *in vivo*, specifically as it relates to how pentobarbital modulates firing frequency and spike timing in each neuron.

Spike firing of the cortical neurons is often analyzed by the firing rate (Tort et al., 2010; Chauvette et al., 2011) and spike correlation, including autocorrelation (Chauvette et al., 2011) and Fourier histograms (Tort et al., 2010), some of which are used to examine the effects of anesthesia (Chauvette et al., 2011). These methods, however, do not necessarily detect the rhythmicity and regularity of neural firing because neural firing often exhibits widely varying interspike intervals (ISIs). The unfolding map, which is used in random matrix theory (RMT) for spectral analysis (Mehta, 1991), defines an average spike interval locally in time and enables the comparison of different activity states on a universal scale. Therefore, the temporal features of action potentials in multiple types of neurons may be described as a simple temporal phenomenon by RMT. RMT was first employed in mathematics and was introduced in physics in the 1950s to describe the energy spectrum of nuclei (Wingner, 1951; Dyson, 1962, 1963a,b). Since then, RMT has been applied not only in nuclear physics but also in statistical and multivariate analyses in many research fields, including biophysics (Akemann et al., 2011). Thus, RMT is an established methodology for analyzing and quantifying randomly occurring events.

In this study, we classified cortical neurons into two subtypes, high frequency with bursting (HFB) neurons and non-HFB neurons, and examined the regularity of spike firing in awake and pentobarbital-induced anesthetized conditions by RMT analysis. We found that HFB neurons, presumably fast-spiking GABAergic (FS) neurons, maintained a more consistent spike firing regularity than did non-HFB neurons. This finding was supported by our *in vitro* experiment that demonstrated smaller variance of the ISI during repetitive spike firing in FS neurons than pyramidal neurons.

## Materials and Methods

All experiments were performed in accordance with the National Institutes of Health Guide for the Care and Use of Laboratory Animals and were approved by the Institutional Animal Care and Use Committee at Nihon University. All efforts were made to minimize the number and suffering of animals used in experiments.

### Animals

Eight-week-old Wistar rats (184.4 ± 16.8 g, *n* = 15, male, Japan SLC) were habituated to a stainless steel cylinder in their home cages (27 cm × 45 cm × 20 cm) in a temperature- and humidity-controlled environment (23 ± 2°C; 55 ± 5%) under a 12-h light/dark cycle with free access to food and water. The rats were handled by an experimenter for 10–15 min twice a day for a week; after habituation, lightweight head attachments (Isomura et al., 2009; Kimura et al., 2012) (Narishige, Tokyo, Japan) were surgically attached to the rat skulls using stainless steel screws and dental resin cement (Super-Bond C&B, Sun Medical, Tokyo, Japan; Unifast III, GC Corporation, Tokyo, Japan) under 2–2.5% isoflurane anesthesia (Pfizer, Tokyo, Japan). The screw set on the frontal cortex (2.5 mm anterior and 2.0 mm lateral to the bregma) was used as an electrode (10 kΩ) for electroencephalogram (EEG) recording. The adequacy of anesthesia was gaged by the absence of the hindlimbpinch reflex. The body temperature was monitored using a rectal probe (BWT-100, Bio Research Center, Japan) and was maintained at approximately 37°C using a heat pad. Rats received analgesic (Carprofen, 5 mg/kg, s.c., Zoetis, Tokyo, Japan) and maintenance medium (10 ml, s.c., Sorita-T3, Ajinomoto, Tokyo, Japan).

*In vitro* patch-clamp experiment was performed using 4-week-old vesicular GABA transporter (VGAT)-Venus line A transgenic rats (Uematsu et al., 2008), which have fluorescent labeling (Venus) of almost all cortical GABAergic cells.

### Behavioral Training

Wistar rats were placed in an automatic task-training system (Isomura et al., 2009; Kimura et al., 2012) (custom-made by O’hara, Tokyo, Japan), and their heads were affixed to the frame. Rats were trained to perform the voluntary forelimb movement task for 2–3 h a day with a 1-h break. A pure-tone cue sound (8 kHz; 1 s) was presented every 4 s while the lever position was at the center. If a rat pushed the spout-lever toward its mouth within 1.5 s of the sound cue, a reward of 15 μl of 5 mM saccharin solution was received. After 3–4 days of training, most rats completed the operant learning task and were able to be kept quiet in the frame. These rats were then used for electrophysiological recording as described below.

### Unit Recording

On a recording day, the trained rat underwent a small craniotomy, and an incision of the dura mater under 2.0–2.5% isoflurane anesthesia was made to insert the recording electrode into the left side of the insula cortex (IC). The IC processes nociception and is considered to integrate nociception with limbic information (Hanamori et al., 1998; Horinuki et al., 2015; Nakamura et al., 2015), and plays a pivotal role in regulating oral functions and integrating interoceptive states into conscious feelings (Naqvi and Bechara, 2009). Ropivacaine hydrochloride (AstraZeneca, Osaka, Japan), a long-lasting local anesthetic, was applied to the incisions to avoid the production of pain after awaking from anesthesia. We secured vascular access from the tail vein with an indwelling needle (Nipro, Osaka, Japan) filled with heparin (200 unit/ml; Wako, Osaka, Japan). After recovery from anesthesia, unit recording was performed.

**FIGURE 1:**
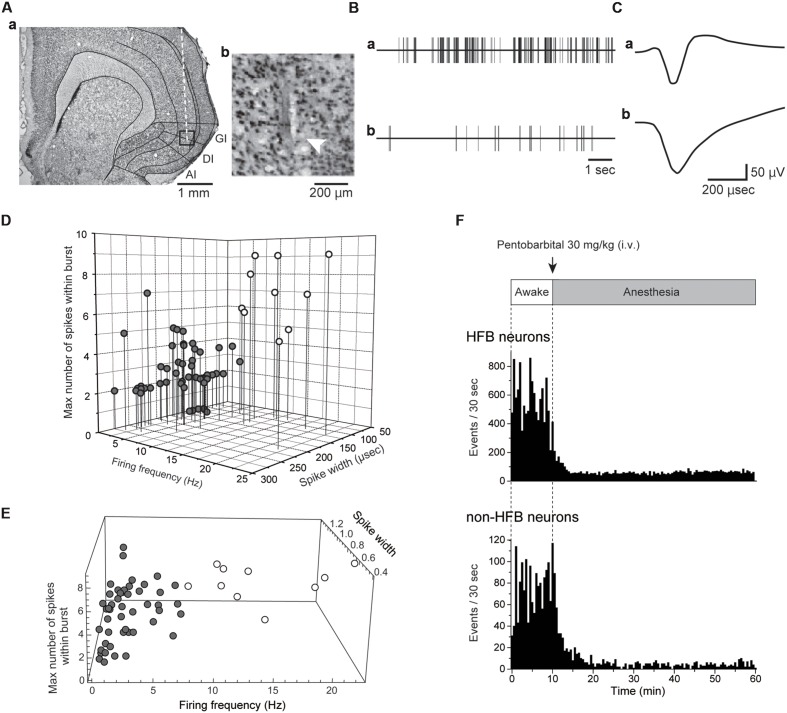
**Extracellular recordings from IC.** **(A)** A coronal Nissl-stained section showing the tract of a recording electrode **(Aa)**. The boxed region in **(Aa)** is magnified in **(Ab)**. The arrowhead indicates the most ventral site of the tract. **(B)** Spontaneous activities of the IC neuron with a high firing rate with a burst **(Ba)** and the IC neurons firing at a low frequency without a burst **(Bb)**. The spikes were truncated. Both activities were recorded under awake conditions. **(C)** Averaged spike waveforms shown in **(B)**. Note the short spike width in **(Ca)** compared with that in **(Cb)**. The neuron shown in **(Ba)** and **(Ca)** is classified as an HFB neuron (see Materials and Methods). **(D)** Distribution pattern of IC neurons in the scatter plot with three axes: firing frequency, spike width, and the maximum number of spikes within the burst (see Materials and Methods). According to the distribution pattern, the IC neurons are divided into two categories: HFB neurons with a high firing frequency (>5 Hz) and large NSB max (≥5; open circles) and non-HFB neurons that fire at low frequency (<10 Hz, gray circles). Most HFB neurons showed a short duration of spikes (<150 μs). **(E)** The result of cluster analysis. The recorded neurons were separated into two groups (open and gray circles) in the same way as **(D)**. **(F)** Spontaneous firing under awake conditions and after pentobarbital injection in the HFB neuron and non-HFB neuron shown in **(Ba), (Ca)** and **(Bb), (Cb)**, respectively. Bin width = 30 s.

The method of unit recording has been described previously (Nakamura et al., 2015). The microelectrode arrays (A1x32-Poly3-10mm-25s-177, NeuroNexus, Ann Arbor, MI, USA), which have 32 circular sensors (diameter = 27 μm, impedance = 1.37 ± 0.1 MΩ at 1 kHz), were perpendicularly inserted 1.0 mm anterior and 5.0 mm lateral to the bregma and 4.0–4.7 mm from the cortical surface (**Figure 1A**). We monitored the spike shapes of all channels on an oscilloscope during the insertion of the electrode and fixed the electrode when spikes were recorded in at least four channels. The number of units recorded from a channel ranged from 0 to 2. *Post hoc* histological examination was performed to identify the site of the electrodes, and units recorded in the channel outside of the IC were not included in further analysis. The action potentials were recorded extracellularly, amplified, filtered, and digitized using a Plexon Recorder System (band pass: 100 Hz–8 kHz; sampling rate 31.25 kHz; Plexon, Dallas, TX, USA) and then stored on a computer hard disk with recording software (ver. 2, Plexon). The spikes were sorted into single units based on the peak amplitude, the sum of the squared amplitude, and the half-width using Off-line Sorter software (ver. 3, Plexon).

Electroencephalograms were recorded by an amplifier (band pass: 1–300 Hz; ER-1, Cygnus Technology, Delaware Water Gap, PA, USA), digitized, and stored on a computer hard disk (Micro 1401 MK2, Cambridge Electronic Design, Cambridge, UK). Power spectrum analysis was performed using dedicated software (NeuroExplorer ver. 4, Plexon).

**FIGURE 2:**
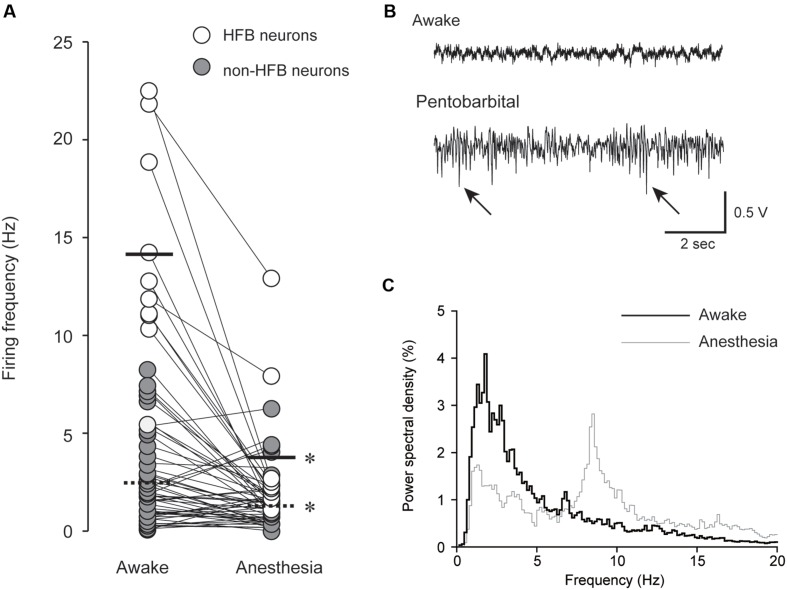
**(A)** Pentobarbital-induced modulation of spontaneous firing frequency in HFB neurons (open; *n* = 10) and non-HFB neurons (gray; *n* = 48). The solid and dashed lines indicate the mean firing frequency of HFB and non-HFB neurons, respectively. Both HFB and non-HFB neurons showed decreased firing frequencies with pentobarbital (^∗^*P* < 0.001, paired *t*-test). **(B)** An example of EEG under awake (upper) and pentobarbital-induced anesthetic conditions (lower). The arrows indicate barbiturate spindles with a predominantly negative polarity. **(C)** Power spectral density analysis obtained from the neuron shown in **(B)**. Note the shift of the peaks (<3 Hz in awake state to 7–8 Hz in anesthetic condition).

After 10–30 min of training, the spontaneously occurring action potentials were recorded for 10–30 min under awake conditions; then, 30 mg/kg pentobarbital sodium (Somnopentyl; Kyoritsu Seiyaku Co., Tokyo, Japan) was intravenously injected via the indwelling needle set in the tail vein as described above. The duration of injection was approximately 30 s. Neural activities gradually changed and reached a steady state within 5 min. We excluded the neurons that showed degrading spike firing. We invariably found barbiturate spindles (Shaw et al., 2001) and an increase in EEG power between 7 and 10 Hz after pentobarbital injection (Noda and Adey, 1973; Jugovac et al., 2006). Pentobarbital-induced increase in burst discharge in the cerebral cortex (Harding et al., 1979; Zurita et al., 1994) is likely to be an underlying mechanism of barbiturate spindle induction. The present EEG finding suggest that our pentobarbital injection protocol reliably induced anesthetic conditions (**Figures 2B,C**). A minimum of ∼100 spikes were required for RMT analysis, but most neurons showed a decreased firing frequency. Therefore, the recording time for anesthetic conditions was extended by 10–30 min beyond that used for awake conditions. During both the awake and anesthetic conditions, recordings were obtained from the same neurons (**Figure 1E**). After recording under anesthetic conditions, the animals were euthanized by inhalation of CO_2_.

**FIGURE 3:**
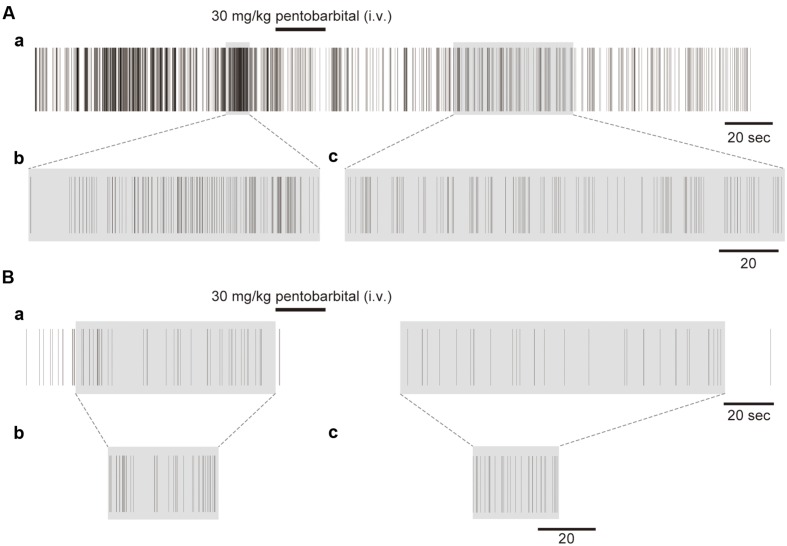
**Examples of the unfolding transformation of spike firing.** **(A)** Spike firing before, during, and after pentobarbital injection **(Aa)**. Pentobarbital decreased the density of spikes. Spike firing with a shadow transformed by RMT analysis is shown in **(Ab)** and **(Ac)**. **(B)** Another example with low spike density **(Ba)**. Shadowed spike firing is transformed by RMT analysis shown in **(Bb)** and **(Bc)**.

### Spike Analysis

The duration at the half-amplitude of the negative peak from the baseline was measured as the spike width. To distinguish burst episodes from isolated spikes, we defined a burst episode as any continuous group of two or more spikes (i) whose minimum ISI was <5 ms and (ii) whose maximum ISI was <2.5 times the minimum ISI. Statistical calculation of spike firing and the power spectral density of spike firing was performed using Neuroexplorer (ver. 4.110, Nex Technologies, USA).

The number of frequency values of Fourier analysis was 512 (bin width = 0.039 ms), with the maximum frequency set to 20 Hz.

### RMT Analysis

Previous studies have demonstrated the advantage of time-rescaling theorem to detect the characteristics of spike firing (Brown et al., 2001). Similar to these methods, we define an average spike interval locally in time and enables us to compare different activity states on a universal scale using RMT analysis, which enables us to distinguish whether events involve regularity. We mapped an experimentally recorded spike train to the spike train of the average interval one at any local time by the unfolding transformation (Wingner, 1951; Brody et al., 1981; Guhr et al., 1998). This procedure removes the system-specific average spike density. We denoted the experimentally obtained train as {*t*_i_}, where {*t*_i_} is the *i*-th spike time, *i* = 1, 2, …, *N*, and *N* is the total number of spikes. We denoted the unfolded spike train as {*x*_i_}. The unfolding transformation and its complete procedure are described in Supplementary Material. The ISI distribution *p(s)* was defined as the probability density for two neighboring spikes, *x*_i_ and *x*_i + 1_, which had the spacing *s*. In cases of an uncorrelated spike train, in which a certain spike does not influence the timing of other spikes, i.e., a randomly sequenced spike train, we showed that *p(s)* becomes the Poisson distribution as follows: The probability that a spike lies between *t* and *t* + *dt* is independent of *t* and is proportional to *dt*. The probability of a spacing *s* is the probability that for a given spike at *t* there is no other spike between *t* and *t* + *s* and that there is a spike between *t* + *s* and *t* + *s* + *ds*. The interval *s* is divided uniformly into *M* parts. The spikes are independent of time. The probability, *p(s;M)*, that there is no spike in *s* is the product of the probabilities that there is no spike in each of the *M* divisions, which is given by

$$p(s;M)=\,{\left(1-\frac{s}{M}\right)}^{M}$$

In the limit, M → ∞, this probability becomes the Poisson distribution,

$$p(s)=e^{-s}$$

*p(s)* expresses the probability of firing at time *s*. The Poisson distribution means that zero interval firing occurs the most often and the probability dampens exponentially as a function of *s*.

Based on this construction, it is clear that any deviation from the Poisson distribution represents a correlation between spikes. The deviation at a specific value of *s* represents the correlation at the time scale *s*.

To characterize correlations, we often use the shape of *p(s)* over a short time, i.e., *s* ∼ 0, and the function of the decrease in *p(s)* over a long time, i.e., *s* → ∞. In the short time scale, if there is a correlation, one of the well-known shapes of *p(s)* includes suppression around *s* ∼ 0 and the appearance of a maximum. Repulsion occurs when the maximum at *s* = 0 in the Poisson distribution has shifted to a finite *s*. For the long time scale, the typical decreases in *p(s)* are power or exponential decays. Exponential decay, which agrees with the Poisson distribution, represents no correlation; however, power decay represents a long-term correlation.

RMT is usually used to extract a universal property of an interacting system. The unfolding transformation removes the system-specific average spike density. Because the unfolding transformation is a coarse-grained operation, other coarse-grained analysis is performed after unfolding to determine the universal nature of a system. For example, the conventional bin width of the unfolded histogram should be sufficiently large. Setting the bin width too small may elicit system-specific nature. The bin width in the RMT is set to the narrowest width that does not exhibit oscillation and/or detailed structure in the histogram. Therefore, we analyzed the decay pattern of the unfolded histogram, which involves coarse-grained but not detailed temporal information, and fitted the decay using an exponential function or power function.

### Whole-Cell Patch-Clamp Recording

Cortical slices including the IC were prepared from VGAT-Venus line A transgenic rats as previously, we reported (Kobayashi et al., 2012; Koyanagi et al., 2014; Yamamoto et al., 2015). Whole-cell patch-clamp recording was obtained from Venus-negative pyramidal neurons and Venus-positive FS neurons identified in layer V by a fluorescence microscope equipped with Nomarski optics (×40, Olympus BX51W1, Tokyo, Japan) and an infrared-sensitive video camera (C3077-78, Hamamatsu Photonics, Hamamatsu, Japan). The composition of ACSF was 126 NaCl, 3 KCl, 2 MgSO_4_, 1.25 NaH_2_PO_4_, 26 NaHCO_3_, 2.0 CaCl_2_, and 10 D-glucose (in mM). Thin-wall borosilicate patch electrodes (2–4 MΩ) were made by a Flaming-Brown micropipette puller (P-97, Sutter Instruments, Novato, CA, USA). The internal solution contained 135 potassium gluconate, 5 KCl, 5 HEPES, 2 MgCl_2_, 2 magnesium ATP, 0.3 sodium GTP, and 5 EGTA in mM. The liquid junction potentials (-13 mV) was not corrected in the present study. Electrical signals were recorded by amplifiers (Multiclamp 700B, Molecular Devices, Sunnyvale, CA, USA), digitized (Digidata 1440A, Molecular Devices), observed on-line, and stored on a computer hard disk using Clampex (pClamp 10, Molecular Devices).

The voltage responses of neurons to long depolarizing current pulse (500 ms) injections were recorded at 30 ± 1°C to examine the ISI during repetitive spike firing. To obtain the variance of the ISIs, 30–50 consecutive responses were recorded in control and during application of pentobarbital sodium (100 μM, Kyoritsu Seiyaku, Tokyo, Japan). The first 10 ISIs were quantitatively analyzed using Clampfit (pClamp 10, Molecular Devices).

### Statistics

Data are expressed as the mean ± SD except as otherwise specified. All data were collected for the RMT analysis and have not been published elsewhere. The imbalance of the sample size between HFB and non-HFB neurons was caused by their population in the IC, as shown in our previous *in vitro* study (Yamamoto et al., 2010). Based on this study, we performed the following statistical tests without any correction. Comparisons of the firing frequency between awake and anesthesia conditions were conducted using a paired *t*-test. Student’s *t*-test was used to compare the firing frequency between HFB and non-HFB neurons. χ^2^ tests were used for statistical comparisons of the occupancy rate of the decay pattern and repulsion between awake and anesthetic conditions. Cluster analysis of the recorded neurons was performed under the condition that the spike width is divided by 200. In the whole-cell patch clamp experiment, the variances of 1st to 10th ISI under pentobarbital application was compared to those in control using a paired *t*-test. The variance of pyramidal neurons was compared with that of FS neurons using Student’s *t*-test.

**FIGURE 4:**
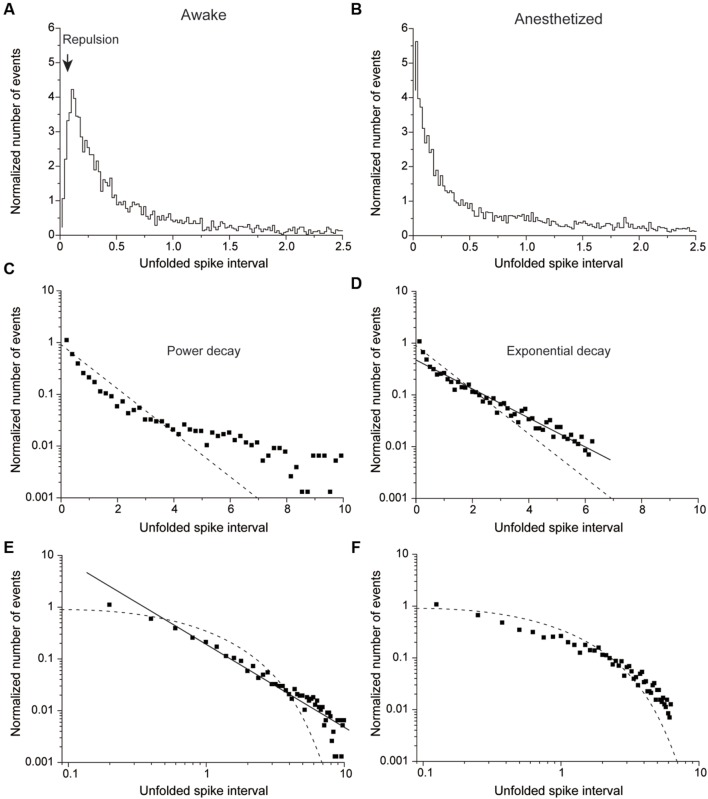
**Normalized number of events plotted against the unfolded spike interval in awake (A,C,E)** and anesthetized conditions **(B,D,F)**. **(A,B)** An example of a non-HFB neuron recorded in awake **(A)** and anesthetized conditions **(B)**. Note repulsion under awake but not anesthetized conditions. **(B)** The unfolded histogram shows recording from the same neuron described in **(A)** in the anesthetized state. Note the disappearance of repulsion. **(C,D)** The same data in **(A)** and **(B)** are plotted on a single logarithmic chart in **(C)** and **(D)**, respectively. **(D)** But not **(C)** is well fitted by a line, which suggests that the distribution pattern of **(D)** was followed an exponential decay. **(E,F)** Plots of a double logarithmic chart shown in **(A)** and **(B)**, respectively. The solid line in **(E)** indicates that the distribution exhibits power decay. The dashed lines in **(C–F)** indicate a Poisson distribution.

**FIGURE 5:**
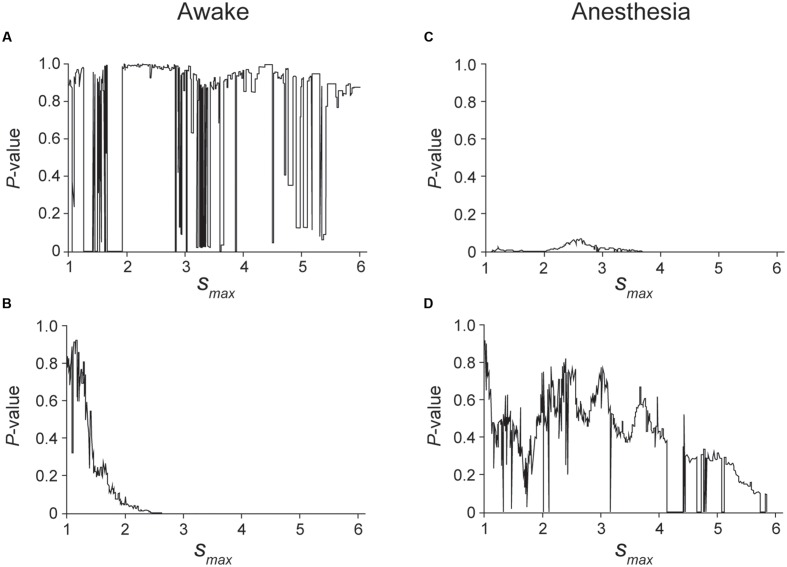
**Kolmogorov–Smirnov (K–S) test was applied to the unfolded histogram shown in **Figure 4** of the awake (A,B)** and anesthetic conditions (**C,D**) to determine which function, a power or an exponential function, is better fitted to the unfolded histograms. The case of larger *P*-value in a power decay than an exponential decay indicates that the unfolded histogram is better fitted by a power decay than an exponential decay, suggesting that the spike firing of this neuron is regulated by preceding spikes. The range of the histogram that was evaluated by the K–S test was set from *s* = 0.5 to *s*_max_. **(A)** *P*-value of the K–S test when the truncated histogram (*s*_max_ was changed from 1 to 6) was fitted by a power function. Although the fitting is unstable in the cases in which 1.3 < *s*_max_ < 1.9, 2.8 < *s*_max_ < 3.6 and 4.7 < *s*_max_ < 5.4, a high *P*-value indicates that the histogram can be fitted by a power function. **(B)** *P*-value of the K–S test when the truncated histogram was fitted by an exponential function. Note that exponential decay was rejected for 2 < s_max_, indicating that an exponential function did not fit the truncated histogram well in the awake condition of this neuron. **(C,D)** *P*-value obtained by fitting the truncated histogram with a power **(C)** or exponential function **(D)**. In the anesthetic condition, the firing of this neuron was better fitted by an exponential function than by a power function.

To determine which function – exponential or power decay – is better fitted to the unfolded histogram, the Kolmogorov–Smirnov test was performed. The function forms are the exponential distribution, *P(s)* = *e*^-λs^, where the parameter is λ, and the type I Pareto distribution, *P(s)* = *(s/k)*^-λ^, where the parameters are *k* and λ. We fitted the data in section *s*_min_ < *s* < *s*_max_⋅ *s*_min_ and *s*_max_ were defined as the minimum and maximum unfolded spike interval that were well fitted by a fitting function, respectively. For example, in **Figures 4** and **5**, *s*_max_ is continuously varied from 1 to 10 in the awake condition and from 1 to 6 in the anesthetized condition to obtain the *P*-value using the Kolmogorov–Smirnov test. We performed parameter fitting using the maximum likelihood method and examined the results obtained using the maximum likelihood logarithmic method. We chose the function whose null hypothesis was not rejected at the 95% confidence level. We adopted the function (power or exponential) that statistically fitted the plot of the unfolded spike interval, if the other function did not fit. In the case in which both functions fit the plot, we evaluated the Classification Index (CI) by comparing the residual sum of squares (RSS) obtained from the following equation:

$$CI=\frac{{RSS}_{\exp}-{RSS}_{pow}}{\frac{\left({RSS}_{\exp}+{RSS}_{pow}\right)}{2}}\times 100$$

**FIGURE 6:**
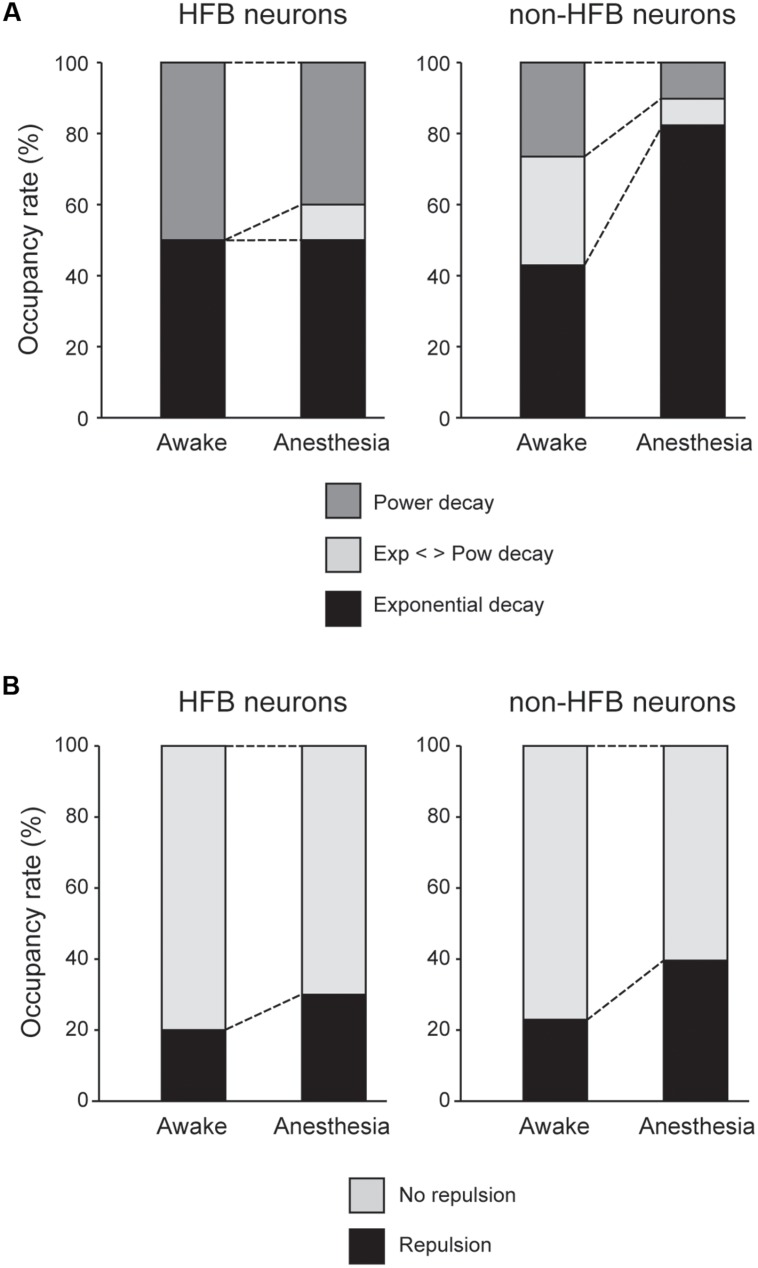
**(A)** Population of HFB and non-HFB neurons fitted with power (dark gray), exponential (black), or intermediate decay (light gray) under awake and anesthetized conditions. Pentobarbital increased non-HFB neurons fitted with exponential decay (*P* < 0.001, χ^2^ test), whereas pentobarbital had little effect on the population of HFB neurons. **(B)** The population of HFB and non-HFB neurons with and without repulsion. Pentobarbital increased the rate of neurons with repulsion in both HFB and non-HFB neurons.

The plot was considered to be fitted by power and exponential functions in the case in which CI > 0.5% and < -0.5%, respectively. In the case in which -0.5% < CI < 0.5%, the plot was considered to fit neither a power nor an exponential function, and the neuron was classified into the intermediate group (light gray, **Figure 6**).

We used original software written in FORTRAN for statistical analyses of the unfolding procedure. The statistical analysis for hypothesis testing was performed using Mathematica (ver. 9.0, by Wolfram Research, Champaign, IL, USA).

Statistical analyses were performed using SPSS (ver. 12.0, Chicago, IL, USA), except for the fitting evaluation and cluster analysis, which was performed with Mathematica (ver. 9.0, by Wolfram Research, Champaign, IL, USA). *P* < 0.05 was considered significant.

## Results

Multiple extracellular recordings were performed from the IC, including granular and dysgranular IC (**Figure 1A**), which receive somatosensory inputs from oral structures (Horinuki et al., 2015; Nakamura et al., 2015). We recorded spontaneous neural activity from 58 neurons under both awake and pentobarbital-induced anesthetic conditions. The recorded neurons were distributed from layers II/III to layer VI. Under awake conditions, the firing frequencies ranged from 0.12 to 22.4 Hz, and the mean frequency was 4.4 ± 5.2 Hz (*n* = 58). The spike duration ranged from 81.3 to 275.0 μs (163.0 ± 52.7 μs, *n* = 58).

### Classification of IC Neurons

As shown in previous *in vitro* patch-clamp studies, FS neurons are the major GABAergic neurons (Markram et al., 2004; Koyanagi et al., 2010; Yamamoto et al., 2010; Kobayashi et al., 2012) and are known to potently inhibit surrounding excitatory neurons (Koyanagi et al., 2014). Our *in vitro* intracellular recordings demonstrate that the spike duration is much shorter in FS neurons than in other cell types (Koyanagi et al., 2010). These electrophysiological features of FS neurons are also applicable in extracellular recordings. *In vivo* juxtacellular recordings, which can confirm the subtype of a recording neuron by *post hoc* histological analysis, reveal that FS neurons show a shorter spike width and a higher baseline frequency than pyramidal neurons (Tseng et al., 2008; Isomura et al., 2009). Recent studies using optogenetics made it possible to activate only parvalbumin-positive (FS) neurons and demonstrated that the spike width of parvalbumin-positive neurons was shorter than that of pyramidal neurons (Cardin et al., 2009; Letzkus et al., 2011). In addition, neurons with high spontaneous firing frequency and narrow spike width often showed burst firing.

With respect to these characteristic features of FS neurons, we classified the neurons recorded as either HFB neurons or non-HFB neurons according to spike parameters, spike width, spike frequency and the number of spikes within a burst (NSB; **Figures 1B–D**). Neurons that had high firing frequencies (>5 Hz) and large NSB max (>5) were classified as HFB neurons (*n* = 10). Neurons with a spike width longer than 150 μs and with firing frequencies lower than 10 Hz were classified as non-HFB neurons (*n* = 48; **Figure 1D**). Thus, HFB neurons were considered to be FS neurons, whereas non-HFB neurons were likely to involve glutamatergic excitatory and non-FS interneurons.

We also performed cluster analysis in reference to adjusted spike width, spike frequency, and the number of spikes within a burst (**Figure 1E**). As a result, the recorded neurons were classified into two groups as same as those in **Figure 1D**, supporting the validity of the classification described above.

### Pentobarbital Suppresses Firing Frequency

Intravenous injection of pentobarbital rapidly decreased the firing frequency of most recorded neurons, and within 10 min, the decreased firing rate reached a plateau. Typical examples of the effects of pentobarbital on spike frequency are shown in **Figure 1F**.

A summary result of the effect of pentobarbital on spontaneous spike firing frequency is shown in **Figure 2A**. In HFB neurons, the firing frequency was reduced from 14.0 ± 5.4 to 3.7 ± 3.8 Hz (*n* = 10, *P* < 0.001, paired *t*-test), and in non-HFB neurons, the firing frequency was reduced from 2.5 ± 2.2 to 1.3 ± 1.3 Hz (*n* = 48, *P* < 0.001, paired *t*-test). The suppression rate of HFB neurons was 73.1 ± 20.6% (*n* = 10), which was significantly larger than that of non-HFB neurons at 13.1 ± 85.2% (*n* = 50; *P* < 0.05, Student’s *t*-test).

### Unfolding Transformation of Neural Firing

**Figure 3A** shows an example of the spike trains before and after the unfolding transformation in the different activity states in a single neuron. **Figure 3Aa** shows spike trains that were experimentally recorded on a real time scale. Both the awake and anesthetic states were involved in the spike firing, with a firing frequency of 12.8 Hz in the awake condition and 1.7 Hz in the anesthetized condition. **Figures 3Ab,c** shows unfolded spike trains on a universal time scale. The real time scale is stretched and diminished locally to make the spike density 1. A high-density region in real time becomes sparse on the universal time scale after the unfolding transformation. **Figure 3B** shows another example with a low firing rate (the firing frequencies were 1.0 Hz in the awake condition and 0.45 Hz in the anesthetized condition). The recorded spikes for 80 and 150 s (**Figure 3Ba**; shadowed spike firing, respectively) were transformed to the short universal time (**Figures 3Bb,c**).

### Estimation of Spike Firing Regularity of RMT Analysis

In cases of no correlation between two adjacent spike timings, the distribution pattern of a plot was fitted by exponential decay without repulsion, whereas in cases of close correlation of spikes, the distribution pattern of a plot should be fitted by a power decay with repulsion (see Materials and Methods). We next analyzed the spike train by unfolding transformation using RMT and examined whether their unfolded histograms had repulsion and which function – exponential or power – fit the histograms.

**Figure 4** shows a typical example of non-HFB neurons whose unfolded histogram was well fitted by a power function in the awake conditions (**Figures 4A,C,E**) and by an exponential function under the pentobarbital application conditions (**Figures 4B,D,F**). In this non-HFB neuron example, repulsion of the histogram was observed in the awake condition (**Figure 4A**) but not in the pentobarbital condition (**Figure 4B**).

### HFB Neurons Maintain High Regularity of Spike Firing in Anesthetic Conditions

The unfolded histogram of non-HFB neurons that fit a power or exponential function occupied 25 and 40% of total non-HFB neurons, respectively, under awake conditions (**Figure 6A**). However, pentobarbital changed this population such that 80% of the non-HFB neurons showed an unfolded histogram that was well fitted by an exponential function, whereas only 10% of the non-HFB neurons were well fitted by a power function (*P* < 0.001, χ^2^ test). In contrast, 50% of HFB neurons showed a unfolded histogram that was well fitted by a power function, and this trend was maintained even after the application of pentobarbital (40%; *P* > 0.5, χ^2^ test).

In contrast to the large difference in the population of unfolded histograms between HFB neurons and non-HFB neurons, the population of HFB neurons with repulsion was similar to that of non-HFB neurons (20 and 23%, respectively; **Figure 6B**). The effect of pentobarbital on the population of neurons with repulsion was also similar in HFB neurons and non-HFB neurons in that the population of neurons with repulsion was increased to 30 and 40% in HFB neurons and non-HFB neurons, respectively.

### FS Neurons Exhibit Smaller Variance of Interspike Interval than Pyramidal Neurons

**FIGURE 7:**
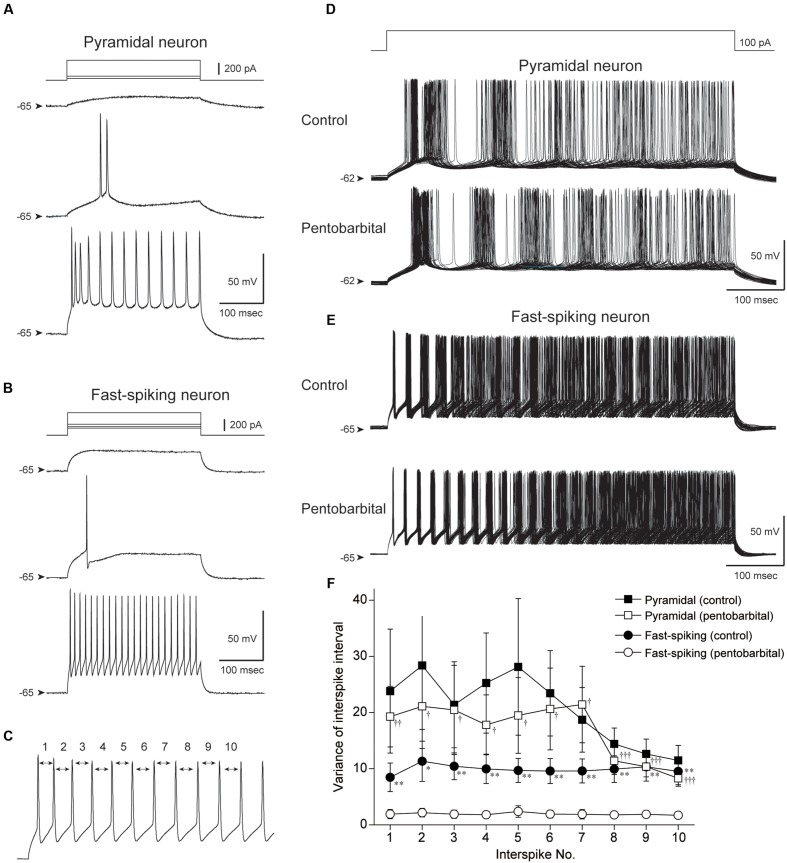
**Comparison of the variance of interspike interval (ISI) between pyramidal and FS neurons obtained by *in vitro* whole-cell patch-clamp recording.** **(A,B)** Spike firing properties of a pyramidal **(A)** and an FS neuron **(B)** responding to depolarizing current pulse injections. Note shorter spike duration and higher repetitive spike firing in the FS neuron. **(C)** The 1st to 10th ISI were evaluated. **(D,E)** Repetitive spike firings of a pyramidal neuron **(D)** and an FS neuron **(E)** responding to 500 ms depolarizing current pulses were superimposed (50 traces). Note the well aligned spikes of the FS neuron in control and under application of 100 μM pentobarbital. **(F)** The variances were plotted against the number of ISI. FS neurons show smaller variance of ISI during pentobarbital application in comparison to those in control (^∗^*P* < 0.05, ^∗∗^*P* < 0.01, paired *t*-test). The variance of FS neurons was smaller than that of pyramidal neurons under pentobarbital application (^†^*P* < 0.05, ^††^*P* < 0.01, ^†††^*P* < 0.001, Student’s *t*-test).

To support the findings obtained from *in vivo* extracellular recording described above, we performed *in vitro* whole-cell patch-clamp recording from Venus-negative pyramidal neurons, whose somata are pyramidal, and Venus-positive FS neurons. FS neurons were characterized by a short spike duration with a large afterhyperpolarization amplitude and a high repetitive spike firing frequency without spike adaptation (**Figure 7B**; Kawaguchi and Kubota, 1997; Kobayashi et al., 2012; Koyanagi et al., 2014). These firing profiles suggest that most of HFB neurons are likely to be FS neurons (Tseng et al., 2008).

Repetitive spike firing was induced by intracellular injection of depolarizing current pulses (500 ms). The current intensity was set at 50–150 pA that induced a constant repetitive firing at 15–40 Hz (**Figures 7A,B**). Under application of ACSF (control condition), pyramidal neurons showed repetitive spike firing with adaptation. The superimposed traces of spike firing exhibited a wide distribution especially in the later part of current injection (**Figure 7D**). This spike jitter is induced by considerably varying ISIs.

On the other hand, spikes of FS neurons showed ordered arrays especially in the early part of current injection (**Figure 7E**). To quantify these findings, the variance of the 1st to 10th ISI was calculated as shown in **Figure 7C**. The variance of each ISI in FS neurons (*n* = 13) tended to be smaller than that in pyramidal neurons (*n* = 16), though it is not statistically significant (*P* > 0.05, Student’s *t*-test).

### Pentobarbital Reduces the Variance of Interspike Interval in FS But Not Pyramidal Neurons

A large part of FS neurons have autapse, which contributes to aligning the timing of spike firing (Bacci and Huguenard, 2006). Therefore, a significantly smaller variance of FS neurons in comparison to pyramidal neurons may be due to the presence of autapse in FS neurons. If so, an facilitating effect of pentobarbital on GABA_A_ receptor-mediated inhibitory post-synaptic currents may further align spike firing in FS but not in pyramidal neurons. To examine this possibility, we evaluated the variance of each ISI before and during bath application of 100 μM pentobarbital in pyramidal and FS neurons.

Pentobarbital had little effect on the variance of the 1st to 10th ISI in pyramidal neurons (*n* = 16, *P* > 0.1, paired *t*-test). In contrast, FS neurons showed a significant decrease in the variance of each ISI during application of pentobarbital (**Figure 7F**; *n* = 13, *P* < 0.01-0.05, paired *t*-test). The findings suggest that spike regularity of FS neurons is maintained even under application of pentobarbital, and fit the above hypothesis obtained from *in vivo* experiments.

## Discussion

The reduction of spontaneous spike firing of IC neurons is a prominent effect of pentobarbital, and the change in spontaneous rate may contribute to anesthesia. The present study used RMT analysis to explore the mechanisms of this finding in detail and extracted the regularity of spike firing, which is regulated by cortical local circuits composed of HFB and non-HFB neurons in addition to their intrinsic membrane properties. We found that during pentobarbital-induced anesthesia, non-HFB neuron firing tended to be randomized, whereas HFB neurons tended to maintain their firing regularity because a spike affected the timing of the next spike. In fact, the superior regularity of ISI in FS neurons, presumably corresponding to HFB neurons, than pyramidal neurons was demonstrated in the present *in vitro* experiment.

### Cell Classification

Because cell classification was performed based on extracellular activities, it is possible that our study reflected the results of contamination from both excitatory and inhibitory neurons in the cerebral cortex. Herein, non-HFB neurons may have included some inhibitory neurons, i.e., regular-spiking and low-threshold spike interneurons. However, 80–90% of neurons in the cerebral cortex are excitatory, and approximately half of all GABAergic interneurons are classified as FS neurons (Kawaguchi and Kubota; 1997). Therefore, we assert that most HFB neurons are FS and that the non-HFB neurons reflect the results obtained from excitatory neurons. Even if interneurons are involved in the non-HFB neuronal responses observed, the percentage of these misclassified interneurons is likely to be small within the non-HFB neuronal population recorded (∼10%).

### Methodological Advantage

Cortical neurons exhibit various temporal firing patterns, such as regular, burst, and chattering, and have wide variations in firing frequency depending on the neuronal subtype and the behavioral state (Koyanagi et al., 2010; Neske et al., 2015). The spike timing in a single neuron has been analyzed by mathematical methods, including autocorrelation and Fourier transformation (Tort et al., 2010; Chauvette et al., 2011). The detection of regularity by both of these methods may be weakened when analyzing spike firing with a mixture of high and low frequencies and/or variable firing patterns.

Random matrix theory analysis normalizes each spike interval by the local average spike frequency and generates spike firing on a universal axis, thus enabling the estimation of spike intervals by absolute values (universal time). The advantages of RMT analysis are characterized by (i) not needing to consider the variation in local spike frequency and (ii) easily estimating how spikes are temporally distributed with respect to the average spike interval. Thus, by RMT analysis, we can understand how a spike influences the induction of subsequent spikes. A spike of a neuron induces synaptic responses in other neurons or itself and may affect the timing of the next spike via local or microcircuits. Therefore, analyzing spike timing enables us to understand the behavior of cortical local circuits, which is critical for elucidating the mechanisms of anesthesia.

The bin size of the unfolded histogram was large enough to exclude the possibility that the absolute refractory period of neurons causes repulsion in the histogram: The absolute refractory period is involved in the bin of the smallest spike interval on a universal time scale. Therefore, the repulsion of the unfolded spike interval histogram is caused not by the neuronal absolute refractory period but by other neuronal mechanisms that delay spike induction, possibly via cortical local circuits.

### Reduction of Firing Frequency by Pentobarbital

Pentobarbital injection consistently decreased the firing rates of both HFB and non-HFB neurons. The decreased firing rate may have been caused by the increase in the open time of GABA_A_ receptors by pentobarbital (Steinbach and Akk, 2001). This conduction change increases the membrane conductance and raises the rheobase (Steinbach and Akk, 2001). Both pyramidal and FS cells receive dense GABAergic inputs from adjacent interneurons in the IC (Koyanagi et al., 2010, 2014; Yamamoto et al., 2010; Kobayashi et al., 2012). Therefore, the increase in GABA_A_-receptor-mediated conductance may induce a stationary reduction in spike frequency. In addition to the enhancement of GABAergic currents via GABA_A_ receptors, pentobarbital has a suppressive effect on AMPA receptors (Taverna et al., 1994; Joo et al., 1999). Pentobarbital-induced suppression of AMPA receptors may also contribute to reducing spontaneous firing frequency both in HFB and non-HFB neurons.

### Why Does the Regularity of Spike Firing of Non-HFB Neurons Decrease under Anesthetic Conditions?

Pyramidal neurons, the principal cortical neurons, clearly exhibit different fluctuations in the membrane potential between the awake (conscious) and unconscious states, which are characterized by “Up states” and “Down states” (Steriade et al., 1993, 2001; Constantinople and Bruno, 2011; Ushimaru et al., 2012). During Up states, membrane potentials are depolarized, which frequently induces action potentials, EEGs exhibit desynchronization (Steriade et al., 2001). In contrast, anesthetic conditions induce alternative changes of the Up and Down states, which show oscillations between the depolarized and hyperpolarized states with a slow-wave EEG (Metherate and Ashe, 1993; Steriade et al., 1993; Cowan and Wilson, 1994).

In cortical local circuits, a spike induced in a pyramidal cell evokes EPSPs in the neurons that receive glutamatergic projections from that cell. Spikes can be elicited by EPSPs in some of these post-synaptic neurons, and the evoked spikes could then influence the pyramidal cell on the generation of the next spike. Regardless of whether these neurons are glutamatergic or GABAergic, the efficacy of inputs from these neurons should be potent during Up states. When the adjacent neurons are glutamatergic, reciprocal glutamatergic inputs facilitate spike generation in the pyramidal cell. However, when the adjacent neurons are GABAergic, the timing of the next spike would be delayed by the returned GABAergic inputs. Thus, the first spike in a pyramidal cell is likely to modulate the initiation of the next spike by activating the adjacent glutamatergic and GABAergic neurons.

In contrast, the influence of the first spike on the second spike is considered to be weakened during Down states because the reversal potential of the GABA_A_-receptor-mediated potential is very close to the membrane potential. Even when a spike is elicited in a pyramidal cell, the adjacent neurons that receive glutamatergic inputs from the pyramidal cell are less likely to fire during Down states. Therefore, the first spike in the pyramidal cell has less influence on the second spike during Down states than during Up states. Considering the longer duration of Down states in the anesthetic condition, the effect of a spike of a pyramidal cell on the initiation of the second spike is larger under awake conditions than under anesthetic conditions. The present findings obtained by RMT analysis support this idea.

In the IC slice preparation, thalamocortical inputs and not a few local connections are lost, and in control condition, most of neurons did not show spontaneous spike firing and rhythmic depolarization of the resting membrane potential, i.e., UP states. Therefore, it is likely that neurons in the IC slice preparation stay in Down states. In the slice experiment, pyramidal neurons exhibited larger variance of ISI than FS neurons. This may support the finding of low regularity of spike firing of non-HFB neurons in Down states.

### Why Does the Regularity of Spike Firing of HFB Neurons Not Change with Pentobarbital?

Pentobarbital decreased regularity in non-HFB neurons but not HFB neurons, although the firing frequency of HFB neurons was significantly decreased by pentobarbital. FS neurons receive glutamatergic inputs from pyramidal cells, and under anesthetic conditions, we discovered that most of these pyramidal cells showed random firing. These findings appear to be inconsistent and raise the question regarding why HFB neurons that receive random excitatory inputs are able to fire with regularity.

Several *in vitro* studies have demonstrated that FS cells fire with a constant ISI via the activation of their autapse (Bacci et al., 2003). More than 50% of FS cells show autaptic currents immediately after action current induction, which suggests that most FS neurons have autapses (Bacci et al., 2003). This reason may explain the higher rate of fluctuation in the histogram fitted to a power function in HFB neurons compared with non-HFB neurons under awake conditions. Pentobarbital may increase autaptic currents in addition to other GABAergic synapses, and this facilitation of autapses could compensate for the decreased regularity of excitatory inputs to HFB neurons.

Our *in vitro* experiment demonstrated a lower variance of ISI in FS neurons than pyramidal neurons in control. This may be a mechanism underlying the regularity of spike firing of HFB neurons. In addition, a decrease in the variance of ISI by application of pentobarbital supports the above hypothesis that facilitation of GABA_A_ receptor-mediated inhibitory synapses including autapses plays a critical role in spike firing regularity in FS neurons.

### Functional Implication

Under awake conditions, at least some pyramidal and FS cells regulate the timing of subsequent action potentials, which results in synchronized outputs from pyramidal cells. In contrast, under pentobarbital-induced anesthetic conditions, FS, but not pyramidal cells, maintain the regulation of spike timing. Taking into account the frequent observation of electrical synapses among FS neurons (Galarreta and Hestrin, 1999; Gibson et al., 1999), the spike firing regulation of FS cells may reset the excitation of cortical circuits and contribute to the induction of slow oscillations. Indeed, pentobarbital increases the EEG frequency of 5–8 Hz (𝜃/α band) as previously reported (Noda and Adey, 1973).

## Author Contributions

All authors had full access to all of the data in the study and take responsibility for the integrity of the data and the accuracy of the data analysis. Study concept and design: MY and MK. Acquisition of data: RK, EY, and MK. Analysis and interpretation of data: RK, MY, EY, NK, and MK. Drafting of the manuscript: RK, MY, and MK. Statistical analysis: RK, MY, NK, and MK. Obtained funding: MK.

## Conflict of Interest Statement

The authors declare that the research was conducted in the absence of any commercial or financial relationships that could be construed as a potential conflict of interest.

The reviewer MBM and handling Editor declared their shared affiliation, and the handling Editor states that the process nevertheless met the standards of a fair and objective review.

## References

[B1] Akemann G. Baik J. Di Francesco P. (2011). *The Oxford Handbook of Random Matrix Theory.* Oxford: Oxford University Press.

[B2] Bacci A. Huguenard J. R. (2006). Enhancement of spike-timing precision by autaptic transmission in neocortical inhibitory interneurons. *Neuron* 49 119–130. 10.1016/j.neuron.2005.12.014 16387644

[B3] Bacci A. Huguenard J. R. Prince D. A. (2003). Functional autaptic neurotransmission in fast-spiking interneurons: a novel form of feedback inhibition in the neocortex. *J. Neurosci.* 23 859–866. 12574414 10.1523/JNEUROSCI.23-03-00859.2003 PMC6741939

[B4] Brody T. A. Flores J. French J. B. Mello P. A. Pandey A. Wong S. S. M. (1981). Random-matrix physics: spectrum and strength fluctuations. *Rev. Mod. Phys.* 53 385–479. 10.1103/RevModPhys.53.385

[B5] Brown R. Barbieri R. Ventura V. Kass R. E. Frank L. M. (2001). The time-rescaling theorem and its application to neural spike train data analysis. *Neural Comput.* 14 325–346. 10.1162/08997660252741149 11802915

[B6] Cardin J. A. Carlén M. Meletis K. Knoblich U. Zhang F. Deisseroth K. (2009). Driving fast-spiking cells induces gamma rhythm and controls sensory responses. *Nature* 459 663–667. 10.1038/nature08002 19396156 PMC3655711

[B7] Chauvette S. Crochet S. Volgushev M. Timofeev I. (2011). Properties of slow oscillation during slow-wave sleep and anesthesia in cats. *J. Neurosci.* 31 14998–15008. 10.1523/JNEUROSCI.2339-11.2011 22016533 PMC3209581

[B8] Constantinople C. M. Bruno R. M. (2011). Effects and mechanisms of wakefulness on local cortical networks. *Neuron* 69 1061–1068. 10.1016/j.neuron.2011.02.040 21435553 PMC3069934

[B9] Cowan R. L. Wilson C. J. (1994). Spontaneous firing patterns and axonal projections of single corticostriatal neurons in the rat medial agranular cortex. *J. Neurophysiol.* 71 17–32. 8158226 10.1152/jn.1994.71.1.17

[B10] Dyson F. J. (1962). Statistical theory of the energy levels of complex systems. I. *J. Math. Phys.* 3 140–156. 10.1063/1.1703773

[B11] Dyson F. J. (1963a). Statistical theory of the energy levels of complex systems. IV. *J. Math. Phys.* 4 701–712. 10.1063/1.1704008

[B12] Dyson F. J. (1963b). Statistical theory of the energy levels of complex systems. V. *J. Math. Phys.* 4 713–719. 10.1063/1.1704009

[B13] el-Beheiry H. Puil E. (1989). Anaesthetic depression of excitatory synaptic transmission in neocortex. *Exp. Brain Res.* 77 87–93. 10.1007/BF00250570 2551715

[B14] Galarreta M. Hestrin S. (1999). A network of fast-spiking cells in the neocortex connected by electrical synapses. *Nature* 402 72–75. 10.1038/47029 10573418

[B15] Gibson J. R. Beierlein M. Connors B. W. (1999). Two networks of electrically coupled inhibitory neurons in neocortex. *Nature* 402 75–79. 10.1038/47035 10573419

[B16] Guhr T. Müller–Groeling A. Weidenmüller H. A. (1998). Random-matrix theories in quantum physics: common concepts. *Phys. Rep.* 299 189–425. 10.1016/S0370-1573(97)00088-4

[B17] Hanamori T. Kunitake T. Kato K. Kannan H. (1998). Responses of neurons in the insular cortex to gustatory, visceral, and nociceptive stimuli in rats. *J. Neurophysiol.* 79 2535–2345. 9582226 10.1152/jn.1998.79.5.2535

[B18] Harding G. W. Stogsdill R. M. Towe A. L. (1979). Relative effects of pentobarbital and chloralose on the responsiveness of neurons in sensorimotor cerebral cortex of the domestic cat. *Neuroscience* 4 369–378. 10.1016/0306-4522(79)90100-3 431818

[B19] Horinuki E. Shinoda M. Shimizu N. Koshikawa N. Kobayashi M. (2015). Orthodontic force facilitates cortical responses to periodontal stimulation. *J. Dent. Res.* 94 1158–1166. 10.1177/0022034515586543 25994177

[B20] Isomura Y. Harukuni R. Takekawa T. Aizawa H. Fukai T. (2009). Microcircuitry coordination of cortical motor information in self-initiation of voluntary movements. *Nat. Neurosci.* 12 1586–1593. 10.1038/nn.2431 19898469

[B21] Joo D. T. Xiong Z. MacDonald J. F. Jia Z. Roder J. Sonner J. (1999). Blockade of glutamate receptors and barbiturate anesthesia: increased sensitivity to pentobarbital-induced anesthesia despite reduced inhibition of AMPA receptors in GluR2 null mutant mice. *Anesthesiology* 91 1329–1341. 10.1097/00000542-199911000-00025 10551584

[B22] Jugovac I. Imas O. Hudetz A. G. (2006). Supraspinal anesthesia: behavioral and electroencephalographic effects of intracerebroventricularly infused pentobarbital, propofol, fentanyl, and midazolam. *Anesthesiology* 105 764–778. 10.1097/00000542-200610000-00023 17006076

[B23] Kawaguchi Y. Kubota Y. (1997). GABAergic cell subtypes and their synaptic connections in rat frontal cortex. *Cereb. Cortex* 7 476–486. 10.1093/cercor/7.6.476 9276173

[B24] Kimura R. Saiki A. Fujiwara-Tsukamoto Y. Ohkubo F. Kitamura K. Matsuzaki M. (2012). Reinforcing operandum: rapid and reliable learning of skilled forelimb movements by head-fixed rodents. *J. Neurophysiol.* 108 1781–1792. 10.1152/jn.00356.2012 22745461

[B25] Kobayashi M. Takei H. Yamamoto K. Hatanaka H. Koshikawa N. (2012). Kinetics of GABAB autoreceptor-mediated suppression of GABA release in rat insular cortex. *J. Neurophysiol.* 107 1431–1442. 10.1152/jn.00813.2011 22190629

[B26] Koyanagi Y. Oi Y. Yamamoto K. Koshikawa N. Kobayashi M. (2014). Fast-spiking cell to pyramidal cell connections are the most sensitive to propofol-induced facilitation of GABAergic currents in rat insular cortex. *Anesthesiology* 121 68–78. 10.1097/ALN.0000000000000183 24577288

[B27] Koyanagi Y. Yamamoto K. Oi Y. Koshikawa N. Kobayashi M. (2010). Presynaptic interneuron subtype- and age-dependent modulation of GABAergic synaptic transmission by β-adrenoceptors in rat insular cortex. *J. Neurophysiol.* 103 2876–2888. 10.1152/jn.00972.2009 20457865

[B28] Letzkus J. J. Wolff S. B. Meyer E. M. Tovote P. Courtin J. Herry C. (2011). A disinhibitory microcircuit for associative fear learning in the auditory cortex. *Nature* 480 331–335. 10.1038/nature10674 22158104

[B29] Markram H. Toledo-Rodriguez M. Wang Y. Gupta A. Silberberg G. Wu C. (2004). Interneurons of the neocortical inhibitory system. *Nat. Rev. Neurosci.* 5 793–807. 10.1038/nrn1519 15378039

[B30] Mehta M. L. (1991). *Random Matrices*, 2nd Edn New York, NY: Academic Press.

[B31] Metherate R. Ashe J. H. (1993). Ionic flux contributions to neocortical slow waves and nucleus basalis-mediated activation: whole-cell recordings in vivo. *J. Neurosci.* 13 5312–5323. 8254377 10.1523/JNEUROSCI.13-12-05312.1993 PMC6576427

[B32] Nakamura H. Kato R. Shirakawa T. Koshikawa N. Kobayashi M. (2015). Spatiotemporal profiles of dental pulp nociception in rat cerebral cortex: an optical imaging study. *J. Comp. Neurol.* 523 1162–1174. 10.1002/cne.23692 25308210

[B33] Naqvi N. H. Bechara A. (2009). The hidden island of addiction: the insula. *Trends Neurosci.* 32 56–67. 10.1016/j.tins.2008.09.009 18986715 PMC3698860

[B34] Neske G. T. Patrick S. L. Connors B. W. (2015). Contributions of diverse excitatory and inhibitory neurons to recurrent network activity in cerebral cortex. *J. Neurosci.* 35 1089–1105. 10.1523/JNEUROSCI.2279-14.2015 25609625 PMC4300319

[B35] Noda H. Adey W. R. (1973). Neuronal activity in the association cortex of the cat during sleep, wakefulness and anesthesia. *Brain Res.* 54 243–259. 10.1016/0006-8993(73)90047-4 4350811

[B36] Patel I. M. Chapin J. K. (1990). Ketamine effects on somatosensory cortical single neurons and on behavior in rats. *Anesth. Analg.* 70 635–644. 10.1213/00000539-199006000-00010 2344058

[B37] Shaw F. Z. Chen R. F. Yen C. T. (2001). Dynamic changes of touch- and laser heat-evoked field potentials of primary somatosensory cortex in awake and pentobarbital-anesthetized rats. *Brain Res.* 911 105–115. 10.1016/S0006-8993(01)02686-5 11511377

[B38] Steinbach J. H. Akk G. (2001). Modulation of GABAA receptor channel gating by pentobarbital. *J. Physiol.* 537 715–733. 10.1113/jphysiol.2001.012818 11744750 PMC2278986

[B39] Steriade M. Nuñez A. Amzica F. (1993). Intracellular analysis of relations between the slow (<1 Hz) neocortical oscillation and other sleep rhythms of the electroencephalogram. *J. Neurosci.* 13 3266–3283. 8340807 10.1523/JNEUROSCI.13-08-03266.1993 PMC6576520

[B40] Steriade M. Timofeev I. Grenier F. (2001). Natural waking and sleep states: a view from inside neocortical neurons. *J. Neurophysiol.* 85 1969–1985. 11353014 10.1152/jn.2001.85.5.1969

[B41] Taub A. H. Katz Y. Lampl I. (2013). Cortical balance of excitation and inhibition is regulated by the rate of synaptic activity. *J. Neurosci.* 33 14359–14368. 10.1523/JNEUROSCI.1748-13.2013 24005289 PMC6618385

[B42] Taverna F. A. Cameron B. R. Hampson D. L. Wang L. Y. MacDonald J. F. (1994). Sensitivity of AMPA receptors to pentobarbital. *Eur. J. Pharmacol.* 267 R3–R5. 10.1016/0922-4106(94)90161-9 8088363

[B43] Thompson S. A. Whiting P. J. Wafford K. A. (1996). Barbiturate interactions at the human GABAA receptor: dependence on receptor subunit combination. *Br. J. Pharmacol.* 117 521–527. 10.1111/j.1476-5381.1996.tb15221.x 8821543 PMC1909313

[B44] Tort A. B. Fontanini A. Kramer M. A. Jones-Lush L. M. Kopell N. J. Katz D. B. (2010). Cortical networks produce three distinct 7-12 Hz rhythms during single sensory responses in the awake rat. *J. Neurosci.* 30 4315–4324. 10.1523/JNEUROSCI.6051-09.2010 20335467 PMC3318968

[B45] Tseng K. Y. Lewis B. L. Hashimoto T. Sesack S. R. Kloc M. Lewis D. A. (2008). A neonatal ventral hippocampal lesion causes functional deficits in adult prefrontal cortical interneurons. *J. Neurosci.* 28 12691–12699. 10.1523/JNEUROSCI.4166-08.2008 19036962 PMC2676938

[B46] Uematsu M. Hirai Y. Karube F. Ebihaara S. Kato M. Abe K. (2008). Quantitative chemical composition of cortical GABAergic neurons revealed in transgenic venus-expressing rats. *Cereb. Cortex* 18 315–330. 10.1093/cercor/bhm056 17517679

[B47] Ushimaru M. Ueta Y. Kawaguchi Y. (2012). Differentiated participation of thalamocortical subnetworks in slow/spindle waves and desynchronization. *J. Neurosci.* 32 1730–1746. 10.1523/JNEUROSCI.4883-11.2012 22302813 PMC6703373

[B48] Vizuete J. A. Pillay S. Diba K. Ropella K. M. Hudetz A. G. (2012). Monosynaptic functional connectivity in cerebral cortex during wakefulness and under graded levels of anesthesia. *Front. Integr. Neurosci.* 6:90 10.3389/fnint.2012.00090 PMC3469825 23091451

[B49] Wakita M. Kotani N. Nonaka K. Shin M. C. Akaike N. (2013). Effects of propofol on GABAergic and glutamatergic transmission in isolated hippocampal single nerve-synapse preparations. *Eur. J. Pharmacol.* 718 63–73. 10.1016/j.ejphar.2013.09.018 24051267

[B50] Wingner E. P. (1951). On the statistical distribution of the widths and spacings of nuclear resonance levels. *Math. Proc. Camb. Philos. Soc.* 47 790–798. 10.1017/S0305004100027237

[B51] Yamamoto K. Koyanagi Y. Koshikawa N. Kobayashi M. (2010). Postsynaptic cell type-dependent cholinergic regulation of GABAergic synaptic transmission in rat insular cortex. *J. Neurophysiol.* 104 1933–1945. 10.1152/jn.00438.2010 20685921

[B52] Yamamoto K. Takei H. Koyanagi Y. Koshikawa N. Kobayashi M. (2015). Presynaptic cell type-dependent regulation of GABAergic synaptic transmission by nitric oxide in rat insular cortex. *Neuroscience* 284 65–77. 10.1016/j.neuroscience.2014.09.062 25286388

[B53] Zurita P. Villa A. E. de Ribaupierre Y. de Ribaupierre F. Rouiller E. M. (1994). Changes of single unit activity in the cat’s auditory thalamus and cortex associated to different anesthetic conditions. *Neurosci. Res.* 19 303–316. 10.1016/0168-0102(94)90043-4 8058206

