# Supplementary material for: Spike Timing Rigidity Is Maintained in Bursting Neurons under Pentobarbital-Induced Anesthetic Conditions
Source: Front Neural Circuits. 2016 Nov 14;10:86. doi: 10.3389/fncir.2016.00086 (PMC5107820; doi:10.3389/fncir.2016.00086)
Supplement: Supplementary file 1 [file Data_Sheet_1.docx]

Supplementary Material

Spike timing rigidity is maintained in bursting neurons under pentobarbital-induced anesthetic conditions

Risako Kato^1,2^, Masanori Yamanaka^3^, Eiko Yokota^1,4^, Noriaki Koshikawa^1,2^,

Masayuki Kobayashi^1,2,5,*^

*** Correspondence:** Masayuki Kobayashi: kobayashi.masayuki@nihon-u.ac.jp

# Unfolding transformation

The method for performing the unfolding transformation is described in previous papers (Wingner, 1951; Brody et al., 1981; Guhr et al., 1998). We denote the experimentally recorded spike train as {*t_i_*}, where *t_i_* is the *i*-th spike time, *i* = 1, 2, …, *N*, and *N* is the total number of spikes. The spectral function of the spike is defined by

|  | $\rho\left( t \right)= \sum_{i=1}^{N} \delta\left( t-t_{i} \right),$ | (S1) |
| --- | --- | --- |

where *δ(t)* is the delta function, which is defined by

|  | $\delta\left( t \right)= \left\{ \begin{aligned} \infty\mathrm{for} t=0 \\ 0 \mathrm{for} t\neq0 \end{aligned} \right. \mathrm{with} \int_{-\infty}^{\infty} \delta\left( t \right)dt=1.$ | (S2) |
| --- | --- | --- |

We define the cumulative spectral function of the spike as

|  | $\eta\left( t \right)= \int_{-\infty}^{t} \rho\left( s \right)ds= \sum_{i=1}^{N} \Theta\left( t-t_{i} \right),$ | (S3) |
| --- | --- | --- |

where $\Theta\left( t \right)$ is the step function, which is defined by

|  | $\Theta\left( t \right)= \left\{ \begin{aligned} 0 \mathrm{for} t<0 \\ \frac{1}{2} \mathrm{for} t=0 \\ 1 \mathrm{for} t>0 \end{aligned} \right. .$ | (S4) |
| --- | --- | --- |

This function counts the number of spikes that fire before or equal to *t* and is also called the staircase function or the number function for spikes. The relationship between *η(t)* and $\rho$*(t)* is

|  | $\rho\left( t \right)= \frac{d\eta(t)}{dt}.$ | (S5) |
| --- | --- | --- |

An example of the cumulative spectral function of spikes is shown in Fig. 7A. Furthermore, it is decomposed into a smooth part, *x(t)*, and a fluctuating part, *f (t)*,

|  | $\eta\left( t \right)=x\left( t \right)+f\left( t \right),$ | (S6) |
| --- | --- | --- |

where the smooth part, *x(t)*, is defined by averaging the cumulative spectral function, where averaging is performed to make the density of the spectral function of the spike reach a value αof one,

|  | $\left\langle\rho(t) \right\rangle=\frac{d\left\langle\eta(t) \right\rangle}{dt}=1,$ | (S7) |
| --- | --- | --- |

and the fluctuating part, *f(t)*, is defined by the difference, *η(t)* − *x(t)*. Here, $\left\langle A \right\rangle$ denotes averaging for an observable variable A. The averaging is defined as

|  | $\left\langle A \right\rangle=\int\int\cdots\int{dt}_{1}{dt}_{2}\cdots{dt}_{N}{AP}_{N}\left( t_{1}, t_{2}, \cdots, t_{N} \right),$ | (S8) |
| --- | --- | --- |

where $P_{N}\left( t_{1}, t_{2}, \cdots, t_{N} \right)$ is the joint probability. The probability that each spike, *t_i_*, in the sector [*t_j_*, *t_j_* + *dt_j_*] is given by $P_{N}\left( t_{1}, t_{2}, \cdots, t_{N} \right){dt}_{1}{dt}_{2}\cdots{dt}_{N}$, where we assume that the probability is normalized and invariant under permutations of the arguments. The *k*-point correlation function $R_{k}\left( t_{1}, t_{2}, \cdots, t_{k} \right)$ is obtained by

|  | $R_{k}\left( t_{1}, t_{2}, \cdots, t_{k} \right)=\frac{N!}{(N-k)!}\int\int\cdots\int{dt}_{k+1}{dt}_{k+2}\cdots{dt}_{N}P_{N}\left( t_{1}, t_{2}, \cdots, t_{N} \right).$ | (S9) |
| --- | --- | --- |

See section 3.1.1 of Ref. Guhr et al., 1998. Analytically, the unfolded variable, $\xi_{i}$, is defined by

|  | $\xi_{i}=\xi_{i}(t_{i})=\int_{-\infty}^{t_{i}} R_{i}(t_{j})dt_{j},$ | (S10) |
| --- | --- | --- |

from which the dependence on the averaged spike density, ${R_{j}(t}_{j})$, has been removed. Using (A8-10), we present the analytical procedure for deriving the unfolded variable, $\xi_{i}$, which is defined in the limit $N\to\infty$. However, we do not know the analytical form of the joint probability function associated with the neuron firing. Therefore, using the similarity between (A3) and (A10), we make an unfolding map from a spike train using (A3) instead of (A8). The smooth part is approximated by a continuous function and is determined by fitting the cumulative spectral function to a higher-order polynomial using, for example, a least-squares method (see Fig.11 and the caption in Ref. Guhr et al., 1998). The cumulative spectral function is obtained by plotting

|  | $\left( t_{n}, n \right),$ | (S11) |
| --- | --- | --- |

where n = 1, 2, · · ·, and the unfolding map is determined by fitting them with a polynomial

|  | $x\left( t \right)=\sum_{i=0}^{N} a_{n}t^{n},$ | (S12) |
| --- | --- | --- |

where *a_n_* are the coefficients estimated by the fitting. In the present study, we used an 8th-order polynomial. Although fitting with a higher-order function shows a better fit to the data, the unfolding function, *x(t)*, must be a monotonically increasing because the chronological order of spikes must not be exchanged before and after the fitting. A decrease in the fitting function means that spike timings are reversed. We performed the fitting from a lower order to a higher order, and the function that is sufficient for both requirements is an 8th order (Fig. 7C). This procedure has been commonly used in the analysis of energy levels in nuclear physics, localization of electrons in solids, quantum chaos, etc.

The unfolded spike train {*x_i_*} is obtained by

|  | $x_{i}=x\left( t_{i} \right)$, *i* = 1, 2, …, *N*. | (S13) |
| --- | --- | --- |

See Fig. 7A. In the unfolded variables, the cumulative spectral function of the spike is

|  | $\tilde{\eta}\left( x \right)=x+ \tilde{f}\left( x \right),$ | (S14) |
| --- | --- | --- |

which, for the spike train, is defined discretely along the *t*-axis,

|  | $\tilde{\eta}\left( x\left( t_{i} \right) \right)=x(t_{i})+ \tilde{f}\left( x\left( t_{i} \right) \right),$ | (S15) |
| --- | --- | --- |

and the corresponding unfolded spike train is defined discretely along the *x*-axis,

|  | $\tilde{\eta}\left( x_{i} \right)= x_{i}+ \tilde{f}\left( x_{i} \right),$ | (S16) |
| --- | --- | --- |

but also for any continuous variables, *t* and *x*, even if there is no spike. The functions marked with a tilde indicate that the argument of the function is the variable after unfolding transformation (Fig. 7B): The unfolding transformation defines a universal time scale continuously. The average spike density of the unfolded spike train, i.e., the derivative of the smooth part with respect to *x*, is unified at any local time. Now, we can verify the measure (= the average spike density is 1) by taking the derivative with respect to *x*.

|  | $\frac{d\tilde{\eta}(x)}{dx}=1+ \frac{d\tilde{f}(x)}{dx}.$ | (S17) |
| --- | --- | --- |

The properties described by equations (A14) and (A17) are shown schematically in Fig. 7B, where we unfold the unfolded spike train, {*x_i_*}. The trend of the double unfolded function is 1. Thus, the local average of spike intervals of all heterogeneous neurons and of different activity states in a single neuron are normalized to one by this procedure, where we should stress that an absolute scale of the fluctuation and the refractory period are also defined.

In the extreme example involving an intensive burst, a long silent interval was usually not analyzed. However, the silent period may be correlated with the burst. It is possible to analyze the whole spike train equivalently by RMT because the means of the spike interval both in the burst and silent parts are normalized to unity.

After the unfolding transformation, we normalize the interspike interval distribution *p(s)* and its first moment to unity,

|  | $\int_{0}^{\infty} p\left( s \right)ds=1,$ | (S18) |
| --- | --- | --- |

and

|  | $\int_{0}^{\infty} sp\left( s \right)ds=1.$ | (S19) |
| --- | --- | --- |

**
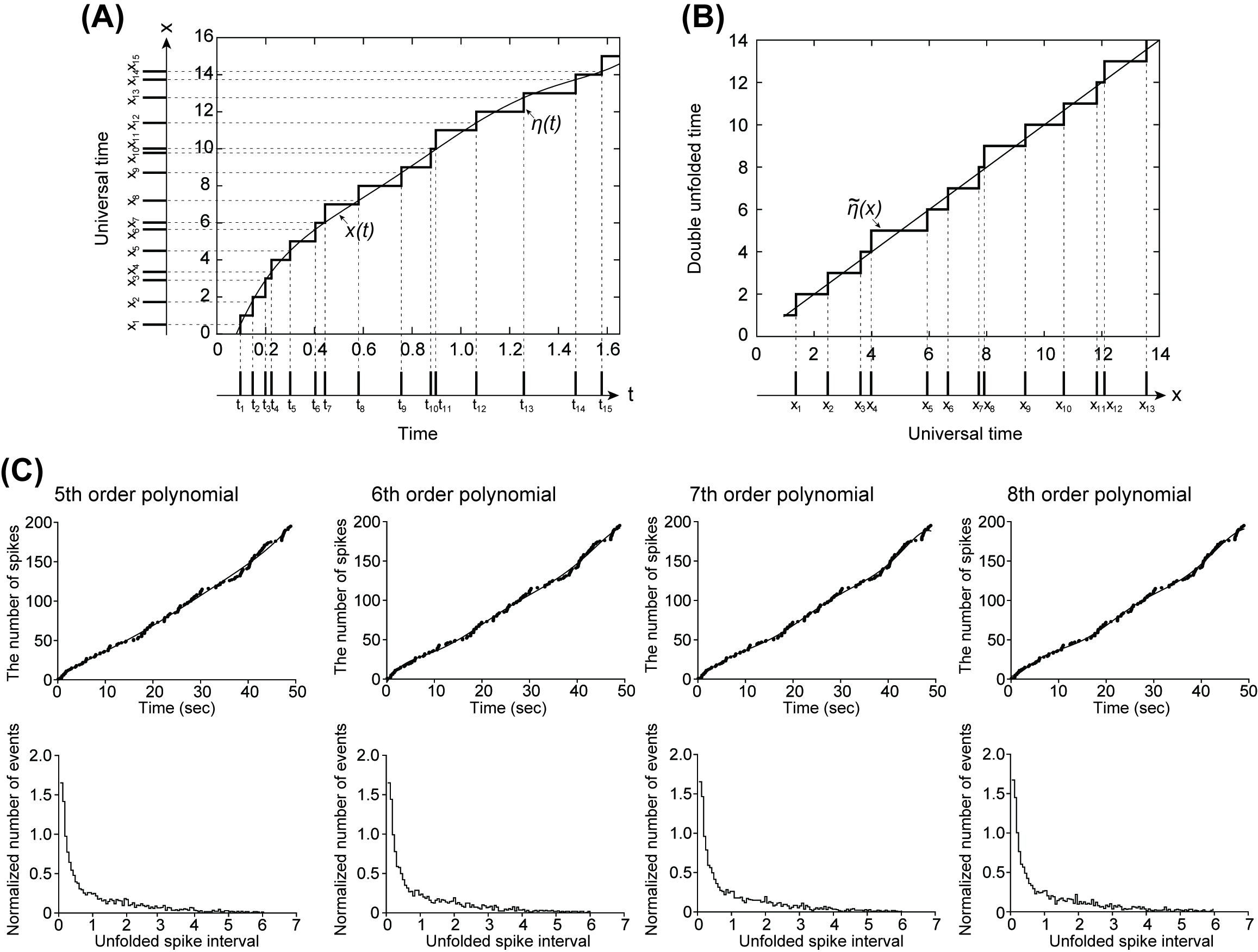
**

**Supplemental Figure 1.** **(A)** Illustration of the actual unfolding procedure. The horizontal axis is the real time axis. A spike train obtained from the experiment is represented by *t_i_*. The cumulative spectral function of the spike, *η(t)*, is represented by the step function. The unfolding map for *t_i_*, *x(t)*, is represented by a curve, which was determined by the least-squares method. The vertical axis is the time axis after unfolding transformation, which represents a universal time scale. The fluctuating part, *f(t)*, is defined by the difference between *x(t)* and *η(t)*. **(B)** Illustration of the unfolded variables by a double unfolding, which explains equations (A14) to (A17). The horizontal axis is the universal time scale obtained by unfolding, i.e., the vertical axis in A. The cumulative spectral function for the unfolded spike, *η(x)*, is represented by the step function. The unfolding map for *x_i_* is represented by a straight line with a slope of 1, which explains the first term on the right-hand side of (A17). **(C)** The upper row shows the fitting of the unfolding maps from 5th- to 8th-order polynomials from left to right. The lower row is the corresponding final histogram of the interspike intervals obtained from the unfolding maps from 5th- to 8th-order polynomials from left to right.
